# Cross-transmission Is Not the Source of New *Mycobacterium abscessus* Infections in a Multicenter Cohort of Cystic Fibrosis Patients

**DOI:** 10.1093/cid/ciz526

**Published:** 2019-06-19

**Authors:** Ronan M Doyle, Marc Rubio, Garth Dixon, John Hartley, Nigel Klein, Pere Coll, Kathryn A Harris

**Affiliations:** 1 Department of Microbiology, Virology and Infection Control, Great Ormond Street Hospital National Health Service Foundation Trust; 2 National Institute for Health Research Biomedical Research Centre at Great Ormond Street Hospital for Children National Health Service Foundation Trust and University College London, United Kingdom; 3 Departament de Genètica i Microbiologia, Universitat Autònoma de Barcelona, Bellaterra, Spain; 4 University College London Great Ormond Street Institute of Child Health, London, United Kingdom; 5 Servei de Microbiologia, Fundació de Gestió de l’Hospital de la Santa Creu i Sant Pau, Barcelona, Spain

**Keywords:** nontuberculous mycobacteria, whole-genome sequencing, transmission, cystic fibrosis, phylogenomics

## Abstract

**Background:**

*Mycobacterium abscessus* is an extensively drug–resistant pathogen that causes pulmonary disease, particularly in cystic fibrosis (CF) patients. Identifying direct patient-to-patient transmission of *M. abscessus* is critically important in directing an infection control policy for the management of risk in CF patients. A variety of clinical labs have used molecular epidemiology to investigate transmission. However, there is still conflicting evidence as to how *M. abscessus* is acquired and whether cross-transmission occurs. Recently, labs have applied whole-genome sequencing (WGS) to investigate this further and, in this study, we investigated whether WGS can reliably identify cross-transmission in *M. abscessus*.

**Methods:**

We retrospectively sequenced the whole genomes of 145 *M. abscessus* isolates from 62 patients, seen at 4 hospitals in 2 countries over 16 years.

**Results:**

We have shown that a comparison of a fixed number of core single nucleotide variants alone cannot be used to infer cross-transmission in *M. abscessus* but does provide enough information to replace multiple existing molecular assays. We detected 1 episode of possible direct patient-to-patient transmission in a sibling pair. We found that patients acquired unique *M. abscessus* strains even after spending considerable time on the same wards with other *M. abscessus–*positive patients.

**Conclusions:**

This novel analysis has demonstrated that the majority of patients in this study have not acquired *M. abscessus* through direct patient-to-patient transmission or a common reservoir. Tracking transmission using WGS will only realize its full potential with proper environmental screening, as well as patient sampling.

## Teaser

Whole-genome sequencing should replace the current molecular typing used routinely in clinical microbiology laboratories. The patient-to-patient spread of *Mycobacterium abscessus* is not common. Environmental screening may provide a better understanding of the acquisition of *M. abscessus* infections.

*Mycobacterium abscessus* (recently renamed as *Mycobacteroides abscessus*) [1], is a group of 3 closely related subspecies: *M. abscessus* subsp. *abscessus*, *M. abscessus* subsp. *massiliense*, and *M. abscessus* subsp. *bolletii* [1, 2]. These rapidly growing, nontuberculous mycobacteria cause chronic pulmonary disease, particularly in patients with cystic fibrosis (CF) and other chronic lung diseases. *Mycobacterium abscessus* is an important pathogen that has emerged in the CF patient population and that has been associated with poor clinical outcomes, especially following lung transplantation [3–5]. This is due, at least in part, to the extensive antibiotic resistance that makes infections with this organism difficult to treat [2, 6]. CF patients infected with *M. abscessus* are frequently not listed for transplant; therefore, the acquisition of this pathogen is considered to be a serious complication in this group.

The epidemiology of *M. abscessus* strains has been studied using Variable Nucleotide Tandem Repeats (VNTR) and Multi Locus Sequence Typing (MLST) [7]. The clustering of globally spread sequence types was confirmed with whole-genome sequencing (WGS) and has provided greater resolution in how the various lineages are related, as well as predicting possible transmission routes [8, 9]. A dominant method of transmission of *M. abscessus* remains contested [10, 11], with evidence for and against patient-to-patient transmission being the common route [8, 12–14]. *Mycobacterium abscessus* is ubiquitous in the environment, with its niche hypothesized to be free-living amoeba [15, 16], but due to the difficulties in isolating the organism, little has been done to track environment-to-patient acquisition. The confirmation of direct patient-to-patient transmission is important, as it influences the management of high-risk patients and it could increase the effectiveness of infection control interventions by directing the use of limited resources.

In this retrospective study, we assessed the utility of using WGS to characterize subspecies, antimicrobial resistance (AMR) profiles, and typing of *M. abscessus* isolates. We also wanted to utilize the data to investigate the scale of patient-to-patient transmission and whether identification of single nucleotide variants (SNVs) by WGS can confirm transmission. To do this, we sequenced the genomes of 145 *M. abscessus* clinical isolates from a well-characterized cohort of 62 patients from 4 hospitals in 2 countries over 16 years.

## METHODS

### Patients and Sample Collection

We collected 33 *M. abscessus* isolates from 30 patients at Hospital de la Santa Creu I Sant Pau (bcn_hsp), Hospital Clínic (bcn_hcl), and Hospital Vall d’Hebron (bcn_hvh) in Barcelona, Spain, and 112 isolates from 32 patients from Great Ormond Street Hospital (GOSH) in London, United Kingdom (Table 1). At GOSH, CF patients were screened for nontuberculous mycobacterial infections when attending clinics, as part of their routine management. In addition to this, CF patients and other patients at all hospitals included in this study were screened for nontuberculous mycobacterial infections when they presented with suggestive clinical symptoms or exacerbations. Demographic and patient location data were obtained from the patient administration system and microbiological data were obtained from the laboratory information management system using Structured Query Language (SQL) and Excel spreadsheets. Additional sources of information included CF and transplant databases. American Thoracic Society consensus guidelines were used to verify evidence of nontubercuolous mycobacterial infections [17]. All investigations were performed in accordance with the Hospitals Research governance policies and procedures.

**Table 1.** Study Patient Information

Patient	Hospital	Subspecies	Sex	Underlying Condition	Source of Isolate	Infection Status at First Contact	Date of First Isolate	Date of First Contact	Date of First Ward Admission
bcn_hcl_002	HCL	*abscessus*	M	Cystic fibrosis	Lung	Already infected	30/12/13	30/12/13	30/12/13
bcn_hcl_004	HCL	*abscessus*	F	Bronchiectasis	Lung	Not infected	09/05/13	01/01/12	01/01/12
bcn_hcl_005	HCL	*abscessus*	F	None	Lung	Already infected	01/08/14	01/08/14	01/08/14
bcn_hcl_007	HCL	*abscessus*	M	Liver neoplasi	Blood	Not infected	10/09/14	2013	2013
bcn_hcl_008	HCL	*massiliense*	M	None	Lung	Already infected	30/06/14	30/06/14	30/06/14
bcn_hcl_009	HCL	*abscessus*	F	Bronchiectasis	Lung	Not infected	08/05/13	2008	2008
bcn_hsp_011	HSP	*bolletii*	M	None	Lung	Already infected	01/12/00	…	…
bcn_hsp_012	HSP	*abscessus*	F	None	Lung	Already infected	31/10/08	31/10/08	31/10/08
bcn_hsp_014	HSP	*abscessus*	F	None	Lung	Already infected	30/01/01	30/01/01	30/01/01
bcn_hsp_019	HSP	*abscessus*	M	None	Lung	Already infected	23/01/14	03/12/04	23/01/14
bcn_hsp_021	HSP	*abscessus*	F	Chronic bronchial infection	Lung	Not infected	24/04/15	02/02/14	02/02/14
bcn_hsp_1	HSP	*abscessus*	F	None	Lung	Already infected	17/09/09	17/09/09	17/09/09
bcn_hsp_2	HSP	*abscessus*	F	None	Lung	Already infected	24/03/09	24/03/09	24/03/09
bcn_hsp_3	HSP	*abscessus*	F	None	Lung	Already infected	05/06/07	05/06/07	05/06/07
bcn_hvh_030	HVH	*abscessus*	F	Cystic fibrosis	Lung	…	09/01/12	…	…
bcn_hvh_031	HVH	*abscessus*	F	Cystic fibrosis	Lung	…	06/05/09	…	…
bcn_hvh_033	HVH	*abscessus*	M	Cystic fibrosis	Lung	…	15/07/13	…	…
bcn_hvh_034	HVH	*massiliense*	F	Cystic fibrosis	Lung	…	23/01/09	…	…
bcn_hvh_035	HVH	*abscessus*	F	Cystic fibrosis	Lung	…	14/02/14	…	…
bcn_hvh_036	HVH	*massiliense*	M	Cystic fibrosis	Lung	…	29/03/11	…	…
bcn_hvh_037	HVH	*abscessus*	F	Cystic fibrosis	Lung	…	30/01/13	…	…
bcn_hvh_038	HVH	*abscessus*	F	Cystic fibrosis	Lung	…	25/07/14	…	…
bcn_hvh_039	HVH	*abscessus*	M	Cystic fibrosis	Lung	…	22/01/07	…	…
bcn_hvh_040	HVH	*massiliense*	M	Cystic fibrosis	Lung	…	04/09/09	…	…
bcn_hvh_041	HVH	*bolletii*	F	Lung transplant	Lung	…	29/08/12	…	…
bcn_hvh_042	HVH	*massiliense*	F	Lung transplant	Lung	…	18/04/12	…	…
bcn_hvh_043	HVH	*abscessus*	M	Lung transplant	Lung	…	23/02/13	…	…
bcn_hvh_045	HVH	*massiliense*	M	Lung transplant	Lung	…	10/04/13	…	…
bcn_hvh_046	HVH	*massiliense*	M	Lung transplant	Lung	…	08/11/13	…	…
bcn_hvh_047	HVH	*abscessus*	F	Lung transplant	Lung	…	28/03/13	…	…
ldn_gos_1	GOSH	*bolletii*	F	Cystic fibrosis	Lung	Already infected	29/06/2004	27/06/2004	27/06/2004
ldn_gos_11	GOSH	*massiliense*	F	Cystic fibrosis	Lung	Not infected	07/10/2008	09/06/1997	15/09/1998
ldn_gos_14	GOSH	*massiliense*	F	Cystic fibrosis	Lung	Already infected	25/04/2005	24/04/2005	24/04/2005
ldn_gos_15	GOSH	*abscessus*	F	Cystic fibrosis	Lung	Not infected	26/06/2006	29/08/1995	26/05/2003
ldn_gos_17	GOSH	*abscessus*	M	Cystic fibrosis	Lung	Not infected	21/04/2009	04/07/1995	01/01/1997
ldn_gos_18	GOSH	*abscessus*	M	Cystic fibrosis	Lung	Not infected	13/12/2004	27/02/2001	03/07/2002
ldn_gos_19	GOSH	*abscessus*	F	Cystic fibrosis	Lung	Not infected	18/06/2007	16/04/1991	27/07/1994
ldn_gos_2	GOSH	*abscessus*	F	Cystic fibrosis	Lung	Already infected	18/08/2005	15/08/2005	15/08/2005
ldn_gos_21	GOSH	*abscessus*	M	Cystic fibrosis	Lung	Not infected	28/10/2008	11/11/1996	20/10/1997
ldn_gos_22	GOSH	*abscessus*	F	Cystic fibrosis	Lung	Not infected	08/05/2008	15/02/1994	05/01/1998
ldn_gos_23	GOSH	*massiliense*	F	Cystic fibrosis	Lung	Not infected	03/02/2009	17/06/2003	10/11/2006
ldn_gos_24	GOSH	*abscessus*	F	Cystic fibrosis	Lung	Already infected	30/03/2009	30/03/2009	30/03/2009
ldn_gos_27	GOSH	*abscessus*	F	Cystic fibrosis	Lung	Not infected	16/10/2010	07/05/2003	07/05/2003
ldn_gos_28	GOSH	*massiliense*	F	Cystic fibrosis	Lung	Already infected	06/06/2011	06/06/2011	06/06/2011
ldn_gos_3	GOSH	*abscessus*	F	Cystic fibrosis	Lung	Already infected	05/09/2005	04/09/2005	04/09/2005
ldn_gos_30	GOSH	*abscessus*	F	Cystic fibrosis	Lung	Not infected	28/06/2012	11/11/1997	13/01/1999
ldn_gos_32	GOSH	*abscessus*	F	Cystic fibrosis	Lung	Already infected	08/11/2010	08/11/2010	08/11/2010
ldn_gos_35	GOSH	*abscessus*	F	Cystic fibrosis	Lung	Not infected	21/10/2013	05/08/2000	05/08/2000
ldn_gos_36	GOSH	*abscessus*	F	CNS tumor	Feces	Not infected	11/01/2014	10/10/2013	N/A
ldn_gos_37	GOSH	*abscessus*	M	Cystic fibrosis	Lung	Not infected	20/02/2014	01/10/2013	28/10/2013
ldn_gos_38	GOSH	*massiliense*	M	Cystic fibrosis	Lung	Already infected	31/07/2014	28/04/2014	28/04/2014
ldn_gos_39	GOSH	*massiliense*	F	Cystic fibrosis	Lung	Already infected	29/09/2014	29/09/2014	29/09/2014
ldn_gos_40	GOSH	*abscessus*	F	Cystic fibrosis	Lung	Already infected	29/03/2015	16/02/2015	16/02/2015
ldn_gos_41	GOSH	*abscessus*	M	Cystic fibrosis	Lung	Already infected	02/06/2015	01/06/2015	01/06/2015
ldn_gos_42	GOSH	*abscessus*	M	SCID	Lung	Not infected	30/07/2015	27/09/2002	…
ldn_gos_43	GOSH	*abscessus*	M	Cystic fibrosis	Lung	Not infected	03/08/2015	02/11/1999	25/02/2001
ldn_gos_44	GOSH	*massiliense*	F	Cystic fibrosis	Lung	Already infected	27/10/2015	26/10/2015	26/10/2015
ldn_gos_45	GOSH	*abscessus*	M	Cystic fibrosis	Lung	Already infected	26/02/2016	05/01/2015	05/01/2015
ldn_gos_46	GOSH	*massiliense*	F	Cystic fibrosis	Lung	Not infected	09/01/2017	10/11/2004	10/11/2004
ldn_gos_7	GOSH	*massiliense*	F	Cystic fibrosis	Lung	Already infected	17/12/2007	17/12/2007	17/12/2007
ldn_gos_8	GOSH	*abscessus*	M	Cystic fibrosis	Lung	Not infected	29/06/2007	17/06/1997	17/06/1998
ldn_gos_9	GOSH	*abscessus*	F	Cystic fibrosis	Lung	Not infected	04/12/2007	20/08/2007	10/06/2008

Abbreviations: CNS, central nervous system; F, female; GOSH, Great Ormond Street Hospital; HCL, Hospital Clínic; HSP, Hospital de la Santa Creu I Sant Pau; HVH, Hospital Vall d’Hebron; M, male; SCID, Severe combined immunodeficiency.

### DNA Extraction, Whole-genome Sequencing, and Multi Locus Sequence Typing

Information on DNA extraction, whole-genome sequencing, and MLST are included in the Supplementary Methods.

### Read Mapping and Variant Calling

Sequenced reads for all samples were first mapped to *M. abscessus* subsp*. abscessus* ATCC 19977 using BBMap v37.90 (Joint Genome Institute). SNVs were called against the reference genome using freebayes v1.2.0 [18], and variants were filtered to only include those at sites with a mapping quality >30, a base quality >30, and at least 5 supporting reads, where the variant was present on at least 2 forward and reverse strand reads and present at the 5’ and 3’ end of at least 2 reads.

### Phylogenetic Analysis

Potential regions of recombination were identified from the consensus genome sequences using Gubbins v2.3.1 [19]. Regions within the genome with low coverage (<5x) were masked on a per sample basis and regions with low coverage across 75% of samples were masked across the entire data set. A maximum likelihood tree was inferred from all samples using RAxML v8.2.4 [20], using a General Time Reversible (GTRCAT) model with 99 bootstraps. Subspecies were identified for each sample based on their position upon this tree.

Separate subtrees were also inferred for *M. abscessus* subsp. *massiliense* sequences, as well as for *M. abscessus* subsp. *abscessus* ST-1 and ST-26 sequences. All samples in each subtree were mapped against a suitable reference. *Mycobacterium abscessus* subsp. *massiliense* str. GO 06 was used as the reference sequencing for study *massiliense* sequences, and the *de novo* assembly of the earliest ST-26 study sequence (ldn_gos_2_520) was used as a reference for other ST-26 samples. *Mycobacterium abscessus* subsp*. abscessus* ATCC 19977 was again used as the reference for ST-1 sequences, as it is the same sequence type. All subtrees were generated using the same method outlined above, apart from the ST-26 subtree, which did not use Gubbins: instead, variants were filtered if 3 SNVs were found within a 100 bp window.

### Sequence Clusters

Sequence clusters to infer possible transmission were generated using 3 different methods on each subtree. First, we used an SNV threshold that was based on the upper bounds of all within-patient diversity and was applied to complete linkage hierarchical clustering based on a pairwise SNV matrix. Secondly, we assigned clusters using the R package rPinecone, as it incorporates SNV thresholds and root-to-tip distances and has been useful when applied to clonal populations [21]. Lastly, we also used hierarchical Bayesian Analysis of Population Structure (hierBAPS) [22] to assign clusters; however, due to the fact that all samples are included in the sequence clusters, we found it was not appropriate for this study question. We made the assumption that any strains taken from different patients that were within-sequence clusters constituted possible transmission events.

### *De Novo* Assembly

All samples underwent *de novo* assembly of bacterial genomes using St. Petersburg genome Assembler (SPAdes) and pilon, wrapped in the Unicycler v0.4.4 package [23]. Assembled contigs were annotated using prokka v1.13 [24] and comparison of the accessory genome was generated using roary v3.12.0 [25]. To generate a list of genes that could be used to differentiate isolates, we filtered the annotated genes to remove coding sequences greater than 8000 bp and less than 250 bp, as well as those only present in a single sample and those present in every sample.

## RESULTS

### *Mycobacterium abscessus* Population Distribution

We obtained WGSs for 145 *M. abscessus* isolates from 62 patients. There were 33 *M. abscessus* isolates from Barcelona, subdivided into 24 *M. abscessus* subsp. *abscessus*, 2 *M. abscessus* subsp. *bolletii*, *and* 7 *M. abscessus* subsp. *massiliense*. There were 112 *M. abscessus* isolates from the United Kingdom, subdivided into 78 *M. abscessus* subsp. *abscessus,* 1 *M. abscessus* subsp. *bolletii*, and 33 *M. abscessus* subsp. *massiliense*. Sample MLST definitions, VNTR definitions, and AMR-associated mutations are shown in Supplementary Table 1.

### Possible Transmission Within *Mycobacterium abscessus* Clusters

**Figure 1.**
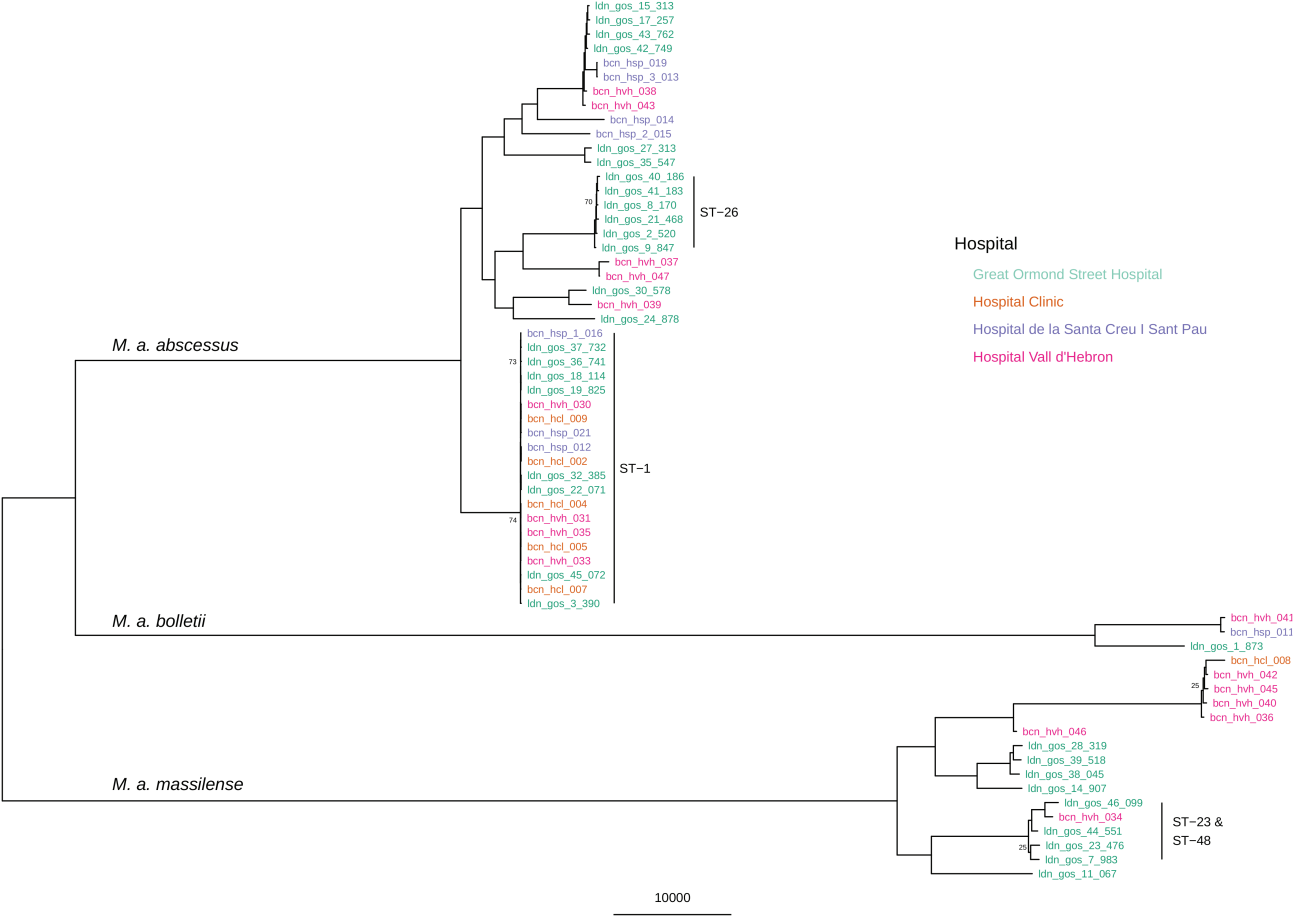
Maximum likelihood SNV tree, using only the earliest isolated sample from all 62 patients. SNVs were identified from mapping reads to the ATCC19977 *Mycobacterium abscessus* subsp. *abscessus* reference genome. Sample names are highlighted in color, based on what hospital they were isolated from: Great Ormond Street Hospital, London, United Kingdom; Hospital Clínic, Barcelona, Spain; Hospital de la Santa Creu i Sant Pau, Barcelona, Spain; and Hospital Vall d’Hebron, Barcelona, Spain. The scale bar represents the number of SNVs and the node bootstrap scores below are shown if below 75. Abbreviation: SNV, single nucleotide variant.

To confirm possible transmission between patients, we required their isolate genomes to be clustered together by 2 independent methods and epidemiological evidence that both patients were at the same hospital during the same time period. Using WGS data, we inferred a phylogenetic tree from a reference genome SNV matrix for all patients (Figure 1). We observed 2 low-variant clusters of isolates that corresponded to ST-1 and ST-26 Pasteur MLST profiles (VNTR II and I, respectively), as well as other closely related *M. abscessus* subsp. *massiliense* isolates between patients. We used an SNV matrix from mapping against a reference (*M. abscessus* subsp. *abscessus* ATCC19977), as well as hierBAPS and rPinecone, to predict sequence clusters. The sequence clusters generated from the single-reference SNV matrix provided no further information than the MLST profiles and, in many cases, provided spurious findings with large groups of isolates clustered with no epidemiological link (Supplementary Figure 1). This included large sequence clusters relating to a single MLST type, which included isolates from different hospitals and countries.

**Figure 2.**
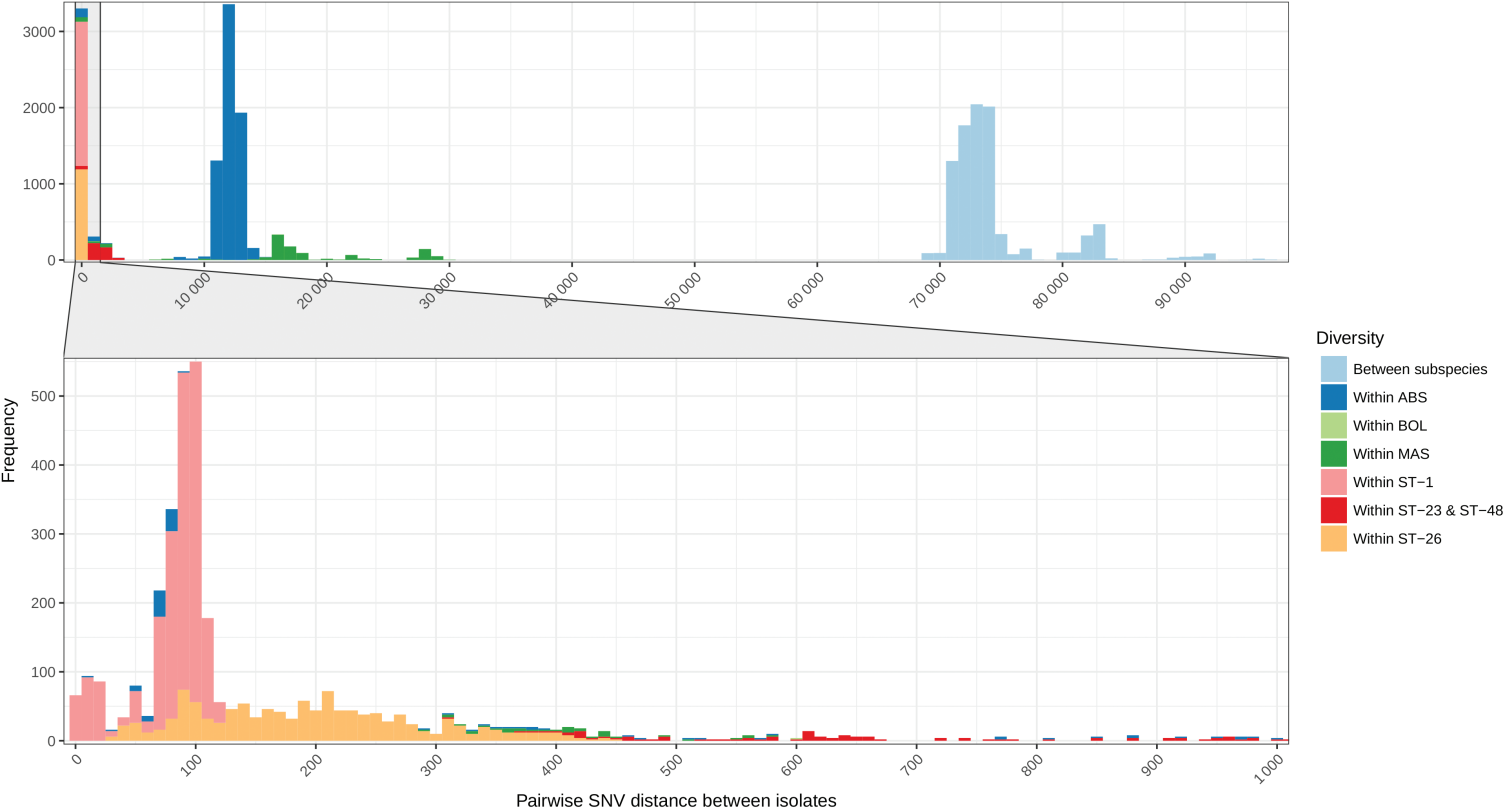
Frequency of pairwise SNV distances between all isolates. SNVs were identified from mapping sequence reads to *Mycobacterium abscessus* subsp. *abscessus* ATCC19977. The full plot includes all samples, while the bottom subsidiary plot only includes isolates that have a pairwise difference between 0 and 1000 SNVs. Abbreviations: ABS; Mycobacterium abscessus subsp. abscessus; BOL, Mycobacterium abscessus subsp. bolletii; MAS, Mycobacterium abscessus subsp. massiliense; SNV, single nucleotide variant; ST, sequence type.

Mapping to a single reference genome led to the inability of a single SNV cut-off, or model, to exclude unrelated isolates from sequence clusters, because the number of pairwise SNV distances varied greatly between both subspecies and specific lineages (Figure 2). For example, the pairwise median SNV distance between just ST-1 isolates was 73 (interquartile range [IQR] 62–81), compared to 29 589 (IQR 27 701–63 703) for all *M. abscessus* subsp. *abscessus* isolates. The same differences were seen in *M. abscessus* subsp. *massiliense*, as well with a pairwise median SNV distance between ST-23 and ST-48 isolates of 2084 (IQR 960–7274), compared to 70 545 (IQR 59 947–71 891) across all isolates from the subspecies.

### Subtree Sequence Clusters

**Figure 3.**
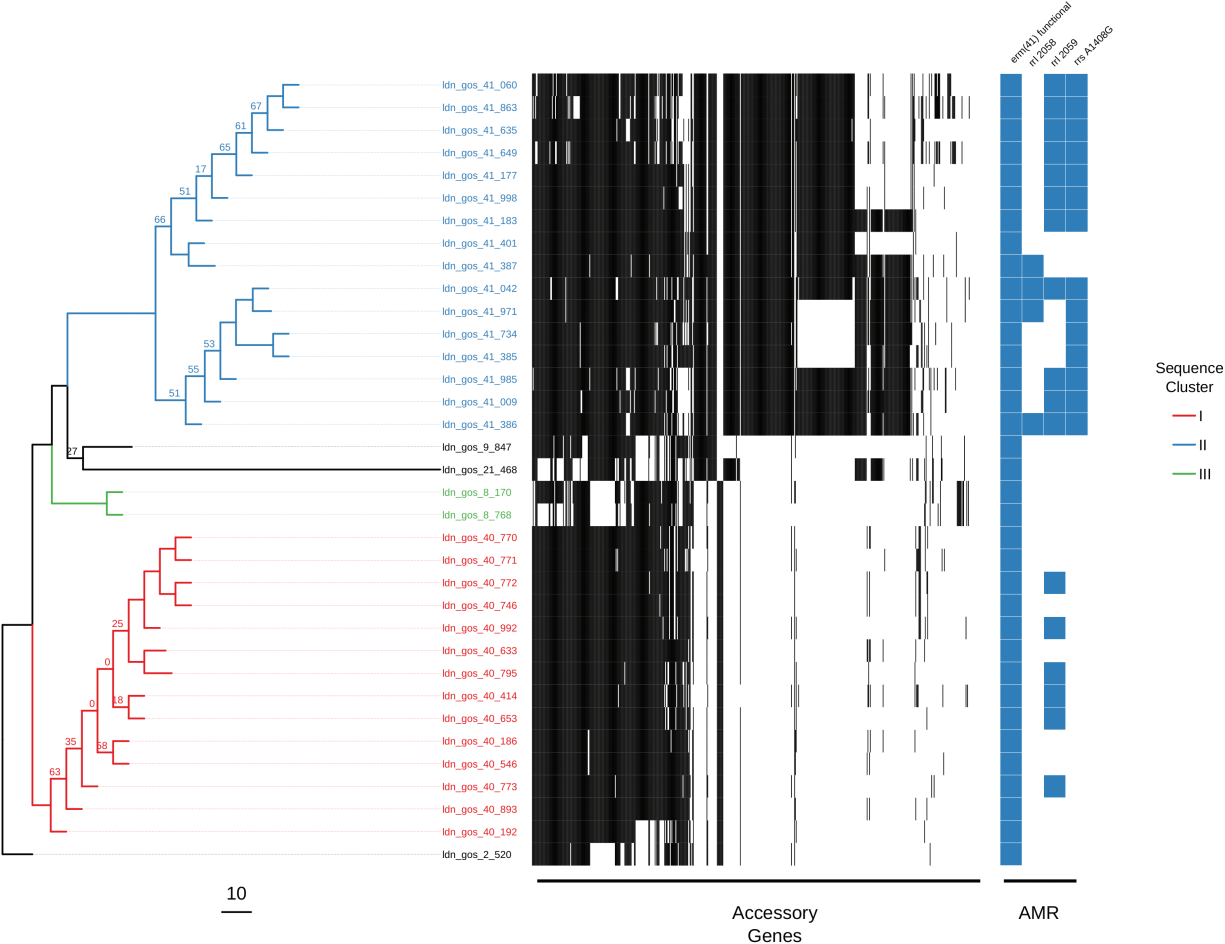
Maximum likelihood SNV tree for all ST-26 isolates. SNVs were identified from mapping reads to a *de novo* assembled study isolate genome (ldn_gos_2_520). Samples are highlighted based on inclusion in sequence clusters. The tree is annotated with the presence (black) and absence (white) of accessory genes, as well as the presence of AMR-associated genes and mutations. This includes the presence of a functional *erm*(*41*) gene conferring inducible resistance to macrolides; the presence of 2 *rrl* mutations conferring high-level macrolide resistance; and the presence of a mutation in *rrs* conferring high-level amikacin resistance. The scale bar represents the number of SNVs and the node bootstrap scores below are shown if below 75. Abbreviations: AMR, antimicrobial resistance; SNV, single nucleotide variant.

**Figure 4.**
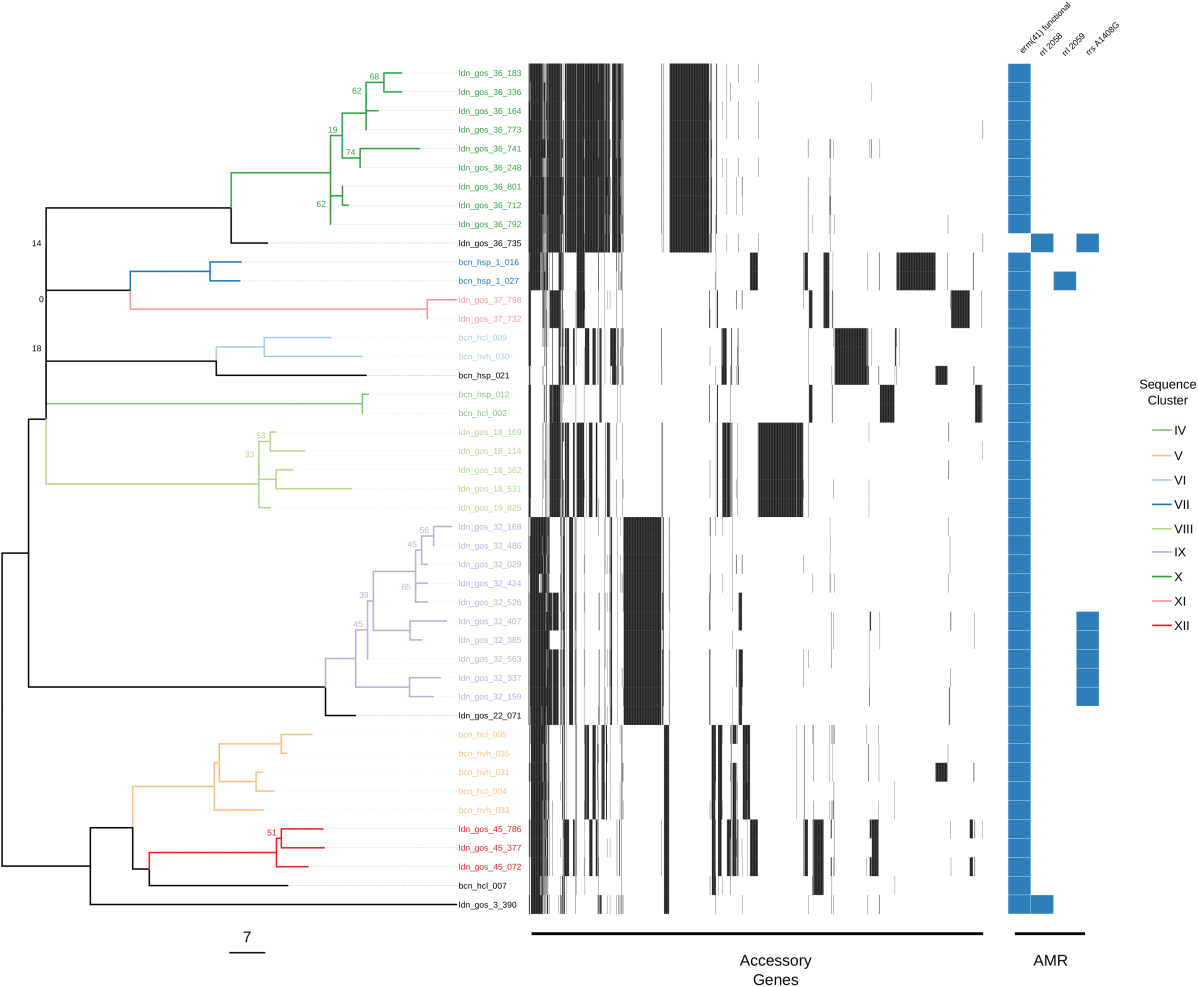
Maximum likelihood SNV tree for all ST-1 isolates. SNVs were identified from mapping reads to *Mycobacterium abscessus* subsp. *abscessus* ATCC19977. Samples are highlighted based on inclusion in sequence clusters. The tree is annotated with the presence (black) and absence (white) of accessory genes, as well as the presence of AMR-associated genes and mutations. This included the presence of a functional *erm*(*41*) gene conferring inducible resistance to macrolides; the presence of 2 *rrl* mutations conferring high-level macrolide resistance; and the presence of a mutation in *rrs* conferring high-level amikacin resistance. The scale bar represents the number of SNVs, and the node bootstrap scores below are shown if below 75. Abbreviations: AMR, antimicrobial resistance; SNV, single nucleotide variant.

The variation in the scale of diversity within subspecies and sequence types hampered efforts to capture possible transmission events. In order to improve the accuracy of sequence clustering, multiple subtrees were made for closely related isolates using a more suitable reference sequence. We separated *M. abscessus* subsp. *abscessus* and *M. abscessus* subsp. *massiliense* isolates, as well as further subtrees for ST-1 (VNTR II), ST-26 (VNTR I), and ST-23/ST-48 (VNTR III) isolates. We also integrated the presence of accessory genes when interrogating possible sequence clusters for transmission (Figures 3, 4, & 5). Sequence clusters were assigned for each subtree using both a single SNV threshold (Supplementary Figure 2) and rPinecone. Overall, we found that predicting transmission from the subtrees reduced the number of different patients clustered together from 46 to 19 and reduced the number of possible sequence clusters suggesting patient-to-patient transmission from 11 to 7.

**Figure 5.**
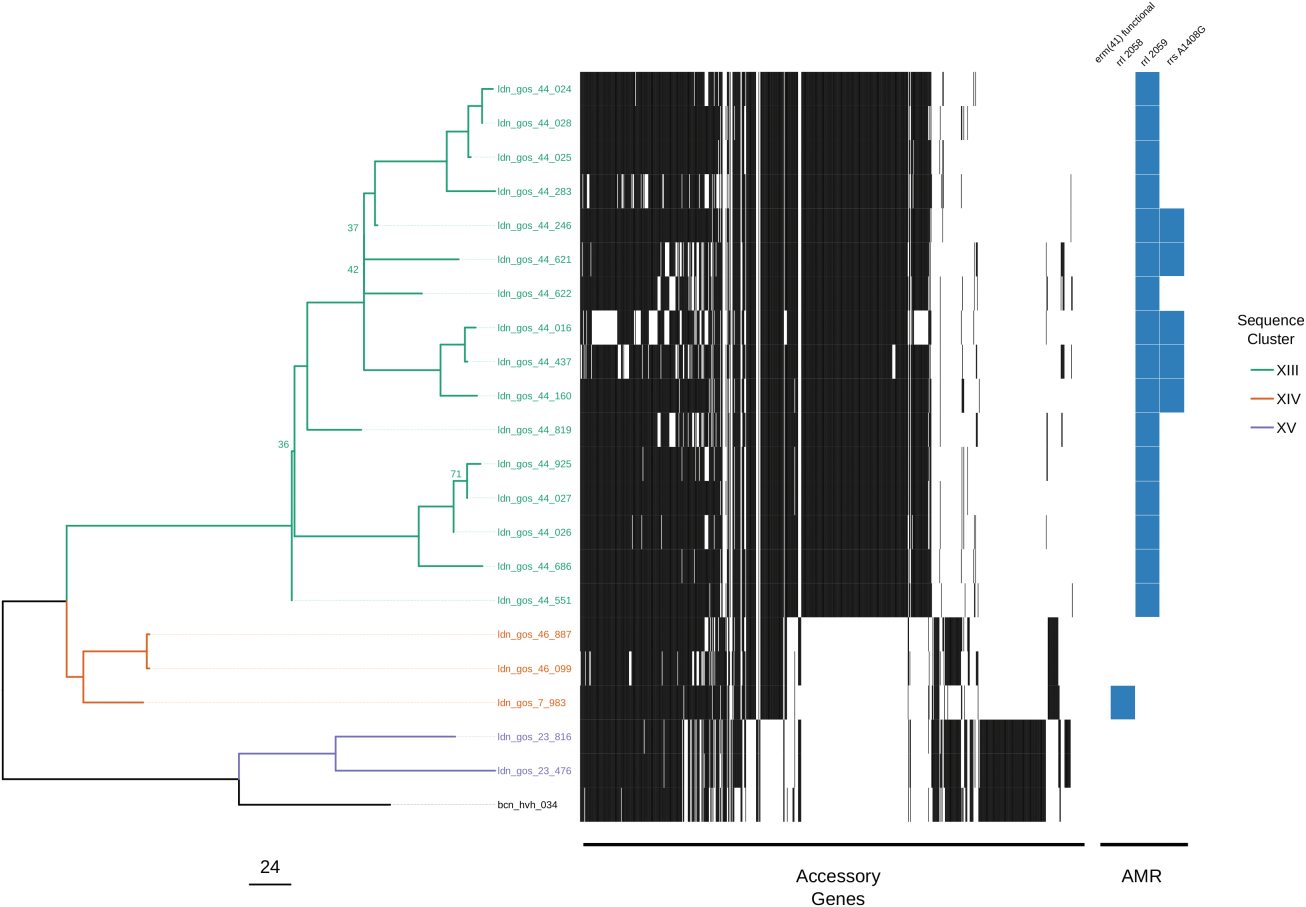
Maximum likelihood SNV tree for all ST-23 and ST-48 isolates. SNVs were identified from mapping reads to *Mycobacterium abscessus* subsp. *massiliense* GO 06. Samples are highlighted based on inclusion in sequence clusters. The tree is annotated with the presence (black) and absence (white) of accessory genes, as well as the presence of AMR-associated genes and mutations. This included the presence of a functional *erm*(*41*) gene conferring inducible resistance to macrolides; the presence of 2 *rrl* mutations conferring high-level macrolide resistance; and the presence of a mutation in *rrs* conferring high-level amikacin resistance. The scale bar represents the number of SNVs and the node bootstrap scores below are shown if below 75. Abbreviations: AMR, antimicrobial resistance; SNV, single nucleotide variant; ST, sequence typing.

A total of 18 sequence clusters (I–XVIII) were identified (listed in Supplementary Table 1): 15 of these were within the subtrees (I–XV), and 7 clusters contained samples from more than 1 patient (IV, V, VI, VIII, XIV, XVI, & XVII). We found no sequence clusters that contained samples from both the United Kingdom and Spain. We found no evidence of transmission between patients within ST-26 (Figure 3). Within ST-1, 4 clusters (IV, V, VI, and VIII) containing samples from more than 1 patient were found. Of these, 3 clusters (IV, V, and VI) contained isolates from 9 patients from multiple hospitals within Barcelona. Only 2 of these patients were in hospital during the same time period (cluster VI: bcn_hcl_009 and bcn_hvh_30), but both were treated in different hospitals. Cluster VIII suggested transmission between 2 patients (ldn_gos_18 and ldn_gos_19) who were siblings and were previously assumed to have been infected either through direct transmission or a common reservoir (Figure 4) [13]. A single cluster (XIV) containing samples from 2 patients (ldn_gos_46 and ldn_gos_7) was found among ST-23 isolates. However, the 2 strains were isolated from samples taken 9 years apart (Figure 5). Patient ldn_gos_7 was already positive for *M. abscessus* on her first admission to GOSH and the 2 patients were present at the lung function lab within a month of each other on 2 occasions, but were never in the same location on the same day and were never admitted to the same ward.

All samples found within their respective clusters also contained similar accessory gene profiles, with the median shared percentage of accessory genes within a sequence cluster being 89% (IQR 79–94%), compared to 18% (IQR 12–37%) for isolates not in the same sequence cluster.

For the 32 GOSH CF patients included in the study, 16 became infected with *M. abscessus* after their first visit to the clinic (Table 1); however, transmission could only be confirmed by both WGS and epidemiological data in 1 case (ldn_gos_19), thus suggesting a different route of acquisition for the rest of these patients.

## DISCUSSION

This study has shown that WGS of *M. abscessus* isolates can determine subspecies, identify previously reported AMR-associated mutations, and provide common typing definitions in a single workflow. This single method can replace the multiple existing molecular assays used in clinical microbiology laboratories to provide the same information and could be used to predict novel resistance variants [26]. We used the WGS data to investigate the likelihood of cross-transmission and found 43 (69%) patients had unique isolates that did not cluster with other patients. We identified 7 sequence clusters from the remaining 19 patients, but only 1 pair of patients (ldn_gos_18 and ldn_gos_19) had a plausible epidemiological link to support possible patient-to-patient transmission, as they were siblings. All other patients with genetically similar strains were either isolated in different countries or different hospitals or were isolated from samples that were taken years apart, making direct transmission of these strains extremely unlikely.

Every *M. abscessus* isolate from a GOSH patient was sequenced, so the data set generated represents a complete picture of *M. abscessus* infection in this hospital, which is vital for inferring transmission. Most of these patients were only attending clinics at GOSH; therefore, this study has captured all of their *M. abscessus* isolates and they are unlikely to have been in contact with *M. abscessus–*positive patients at other hospitals (Table 1). Therefore, if direct patient-to-patient transmission was occurring frequently, we would expect to see evidence of it here. In contrast to this, we found that the majority of patients in this study had unique strains and the majority of sequence clusters were multiple isolates from the same patients. This study confirms previous findings that, despite many *M. abscessus–*negative patients spending considerable time on the same wards as patients with ongoing *M. abscessus* infections, they did not subsequently acquire genetically similar isolates [13, 14, 27].

We have, therefore, found that a fixed number of SNVs cannot be reliably used to infer cross-transmission across all *M. abscessus* isolates, as there seem to be irreconcilable differences in the substitution rate between both subspecies and dominant clones. These difficulties are similar to those seen in *Legionella pneumophila* outbreaks, where the majority of cases can belong to only a few sequence types [25]. *Legionella pneumophila* can also display different scales of genetic diversity within different sequences or genotypes, indicating that a single SNV threshold cut-off will not provide sufficient discriminatory power [26]. When using WGS to infer relatedness in *M. abscessus*, there has previously been an attempt to find an absolute threshold which can rule in or rule out strains in a transmission event. This has previously been placed as below 25–30 SNVs [8, 14, 28, 29]. From our findings, we would advocate using a suitable, genetically similar reference sequence when carrying out core genome SNV calling, especially for the dominant clones, such as ST-1 and ST-26. There is a large amount of variation within the genomes of *M. abscessus* [30], so the use of a single reference, such as *M. abscessus* subsp. *abscessus* ATCC 19977, will mask many differences between strains and generate spurious clusters of genetically similar sequences. Where a suitable reference is not available, we recommend using a high-quality draft *de novo* assembly of the first isolated sample to compare other isolates against, as in the example of the ST-26 samples in this study (Figure 3).

In addition to conducting the core genome SNV analysis, we also found that the integration of accessory genome information is a useful indicator of relatedness within *M. abscessus* isolates that can be used to further interrogate assigned sequence clusters. Generally, there was good concordance between the proportion of putative genes shared and the SNV distance between 2 samples. This was helped by using closely related reference sequences to map sequence reads against. We have seen in this study and previously [31] that there is diversity in the accessory genome profiles—as well as in the number of SNPs and AMR-associated mutations—taken from multiple samples from the same patient on the same day. However, we have always found interpatient diversity to be greater than that seen within the same patient. This would suggest that any direct transmission between patients of even minority populations would still be identified by WGS and, taken together, the data suggest that person-to-person transmission of *M. abscessus* in pediatric patients in our institution is very uncommon. In this study, we have an example of 2 patients with transmission predicted by genomic epidemiology (ldn_gos_7 and ldn_gos_46) that had attended a lung function laboratory on 3 occasions within a month of each other. In this case, the only way transmission could have occurred would be if ldn_gos_7, who was already infected, contaminated the environment and the infection was then transmitted to ldn_gos_46. The predominant view [8] that human-to-human transmission occurs via contamination of fomites by respiratory secretions could explain this, although no other instances of this appeared to have occurred, despite numerous other CF patients attending the unit over many years. What is harder to explain is that, for this to be the case, the interval between exposure and culture positivity was 9 years. It could be that *M. abscessus* remains present but undetectable by conventional methods for this time period or, intriguingly, could cause a latent infection, like what occurs with *Mycobacterium tuberculosis*. To the best of our knowledge, this has never been a demonstrated part of the pathogenesis of *M. abscessus* infection, and may be worthy of further investigation.

In agreement with previous studies, we found an international distribution of *M. abscessus–*dominant clones [8]. We found WGS to be useful to confirm whether different patients’ strains are unrelated, even within the dominant clones, but it has been far more difficult to reach definite conclusions about cross-transmission. Without environmental samples, we cannot rule out the possibility of intermediate sources of infection; therefore, WGS as a tool for tracking cross-transmission in *M. abscessus* will only realize its full potential with the proper screening of environmental sources, alongside longitudinal patient sampling.

## Supplementary Data

Supplementary materials are available at *Clinical Infectious Diseases* online. Consisting of data provided by the authors to benefit the reader, the posted materials are not copyedited and are the sole responsibility of the authors, so questions or comments should be addressed to the corresponding author.

ciz526_suppl_Supplementary_Figure_S1

ciz526_suppl_Supplementary_Figure_S2

ciz526_suppl_Supplementary_Legends

ciz526_suppl_Supplementary_Methods

ciz526_suppl_Supplementary_Table_1

## Notes

***Acknowledgments.*** The authors thank the Biomedical Scientist team for sample collection at Great Ormond Street Hospital, as well as Dr Julià Gonzalez and Dr Teresa Tórtola for sample collection at Hospital Clinic and Hospital de la Vall d’Hebron, respectively.

***Financial support.*** This work was supported by the National Institute for Health Research (European Molecular Biology Organization Short-Term Fellowship 7307 to M. R.) and the European Association of National Metrology Institutes (grant number 15HLT07 to R. M. D.).

***Potential conflicts of interest.*** The authors report no potential conflicts. All authors have submitted the ICMJE Form for Disclosure of Potential Conflicts of Interest. Conflicts that the editors consider relevant to the content of the manuscript have been disclosed.
