# Supplementary figures and images for: Cross-transmission Is Not the Source of New Mycobacterium abscessus Infections in a Multicenter Cohort of Cystic Fibrosis Patients
Source: Clin Infect Dis. 2019 Jun 19;70(9):1855–64. doi: 10.1093/cid/ciz526 (PMC7156781; doi:10.1093/cid/ciz526)

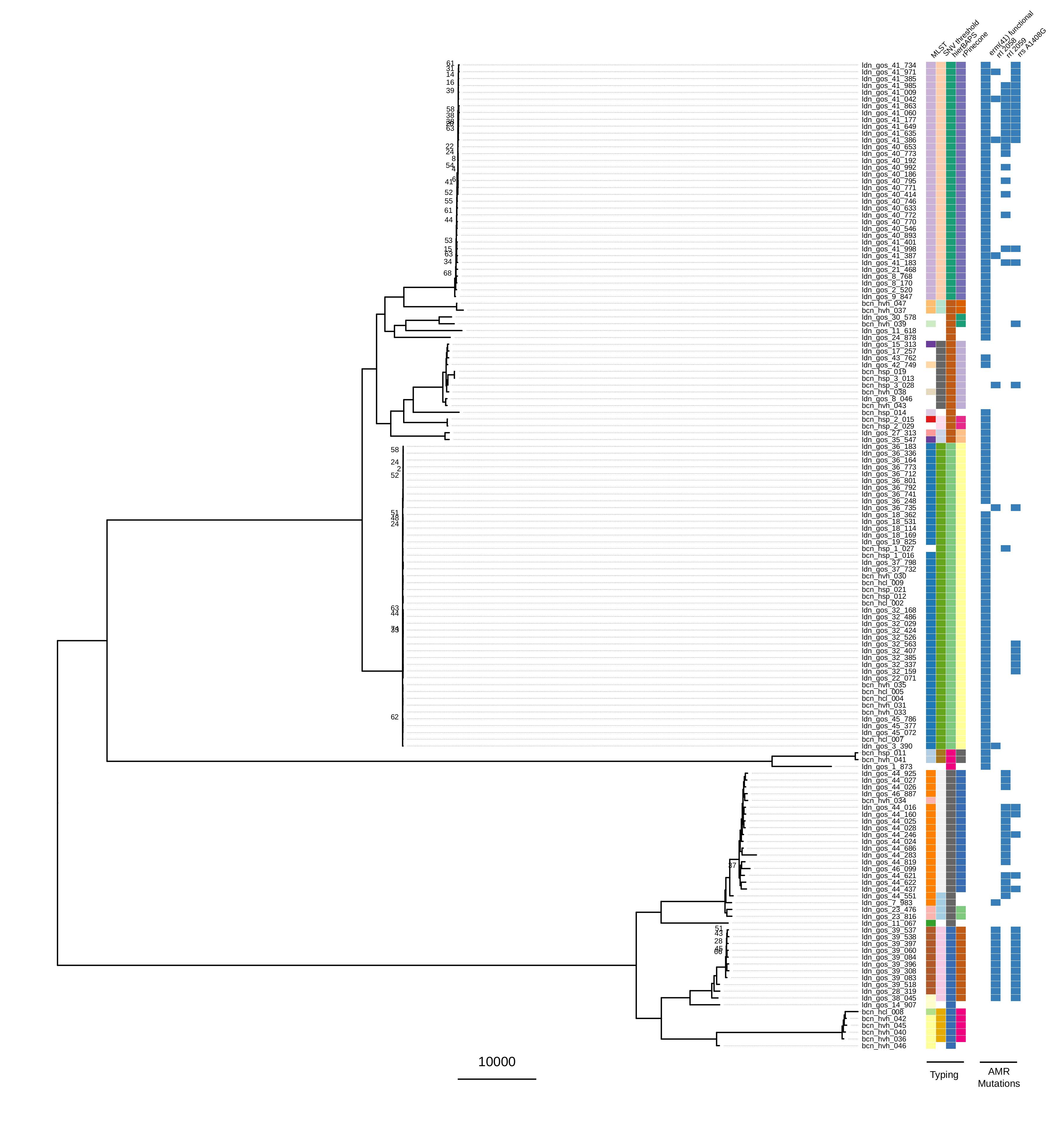

Supplement: ciz526_suppl_Supplementary_Figure_S1 [file ciz526_suppl_supplementary_figure_s1.png]

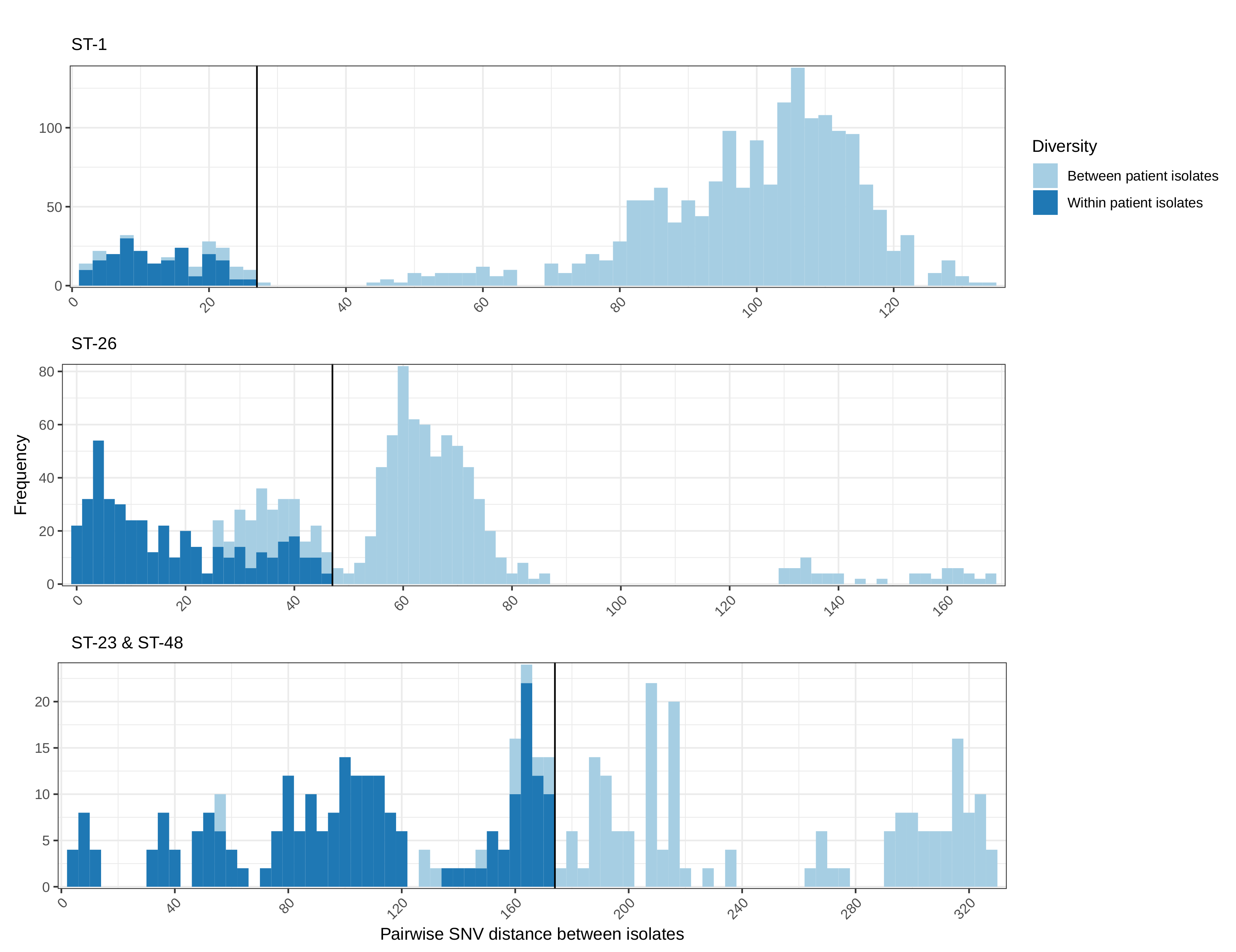

Supplement: ciz526_suppl_Supplementary_Figure_S2 [file ciz526_suppl_supplementary_figure_s2.png]
