# Supplementary material for: Cross-transmission Is Not the Source of New Mycobacterium abscessus Infections in a Multicenter Cohort of Cystic Fibrosis Patients
Source: Clin Infect Dis. 2019 Jun 19;70(9):1855–64. doi: 10.1093/cid/ciz526 (PMC7156781; doi:10.1093/cid/ciz526)
Supplement: ciz526_suppl_Supplementary_Legends [file ciz526_suppl_supplementary_legends.docx]

**Supplementary material**

**Supplementary Methods.**

**Supplementary table 1. Information on all individual *M. abscessus* isolates included in this study.**

**Supplementary Figure 1.** **Maximum likelihood single nucleotide variant (SNV) tree for all isolates in this study.** The tree is annotated with sequence clusters that are defined either by (from left-to-right) MLST, SNV threshold, hierBAPS and rPinecone as well as the presence of AMR associated gene and mutations. This included presence of a functional *erm(41)* gene conferring inducible resistance to macrolides, presence of two *rrl* mutations conferring high level macrolide resistance and the presence of mutation in *rrs* conferring high level amikacin resistance. The scale bar represents the number of single nucleotide variants and node bootstrap scores below are shown if below 75.

**Supplementary Figure 2. Frequency of pairwise single nucleotide variant (SNV) distances between samples after sub-tree analysis**. Figure 2A shows pairwise differences from the ST-1 subtree. Figure 2B shows pairwise differences from the ST-26 subtree. Figure 2C shows pairwise differences from the ST-23 and ST-48 subtree.
