# Supplementary material for: Cross-transmission Is Not the Source of New Mycobacterium abscessus Infections in a Multicenter Cohort of Cystic Fibrosis Patients
Source: Clin Infect Dis. 2019 Jun 19;70(9):1855–64. doi: 10.1093/cid/ciz526 (PMC7156781; doi:10.1093/cid/ciz526)
Supplement: ciz526_suppl_Supplementary_Methods [file ciz526_suppl_supplementary_methods.docx]

**Supplementary Methods**

**DNA extraction and Whole-Genome Sequencing**

One hundred and forty-five *M. abscessus* isolates from 62 patients were analysed using whole-genome sequencing. Briefly, DNA was extracted from all isolates as previously described [18]⁠⁠ with some modifications: DNA was extracted from all isolates using Qiagen EZ1 Blood extraction kit with a previous step of bead-beating (Qiagen, Crawley, United Kindom). Then total DNA concentration was determined using a Qubit fluorometer (Thermofisher). Fifty nanograms of DNA was prepared using Nextera Library Preparation kit (Illumina) and post-PCR clean-up was carried out using Ampure XP beads (Beckman). Library size was validated using the Agilent 2200 TapeStation with Agilent D5000 ScreenTape System (Willoughby, Australia) and 150bp paired-end reads were sequenced on a NextSeq 550 system (Illumina). Raw sequencing reads have been deposited on ENA (study accession PRJEB31559).

**Multi Locus Sequence Typing (MLST) analysis**

We used a custom bash script to extract the alleles of the multi-locus sequence typing (MLST) profile from the mapped reads to the reference genome. The MLST profile was obtained using the Institut Pasteur MLST database ([http://bigsdb.pasteur.fr/mycoabscessus/mycoabscessus.html)](http://bigsdb.pasteur.fr/mycoabscessus/mycoabscessus.html)(17).
